# Disparities in COVID-19 Related Worry Among Older Adults in the United States

**DOI:** 10.1093/geroni/igab046.1859

**Published:** 2021-12-17

**Authors:** Felicia Wheaton, Terika Scatliffe, Matilda Johnson

**Affiliations:** 1 Xavier University of Louisiana, New Orleans, Louisiana, United States; 2 Bethune-Cookman University, Daytona Beach, Florida, United States

## Abstract

Racial disparities in COVID-19 exposure, illness, hospitalization and mortality have been well-documented, however, less is known about whether African Americans and other minorities experience greater worry related to the COVID-19 pandemic. Data from the special midterm release of the 2020 Health and Retirement Study (HRS) were used to examine the relationship between race (white, African American, and other) and ethnicity (Hispanic/Non-Hispanic) and COVID-19 related worry among older Americans (N=2,069). Participants were asked, “because of the coronavirus pandemic how worried are you about 1) your own health, 2) the health of others in your family? 3) Your financial situation? 4) Being able to get help if you needed it from family, friends, or others? 5) What will happen in the future?” (0=not at all worried and 10=very worried). Results from OLS regression controlling for age, gender and education showed that compared with whites, African Americans had significantly higher average worry for all items except the last (other race did not differ). On the other hand, Hispanics had significantly lower worry, on average, for each of the five items. In addition, women had significantly higher average worry, while age was negatively associated with all items except the first. These findings indicate that in addition to the previously documented disparities in COVID-19, older African Americans experienced more worry. This has important implications for long-term physical and mental health.

